# Promoting Functional Activity Engagement in People with Multiple Disabilities through the Use of Microswitch-Aided Programs

**DOI:** 10.3389/fpubh.2017.00205

**Published:** 2017-08-10

**Authors:** Giulio E. Lancioni, Nirbhay N. Singh, Mark F. O’Reilly, Jeff Sigafoos, Gloria Alberti, Viviana Perilli, Francesca Campodonico

**Affiliations:** ^1^University of Bari, Bari, Italy; ^2^Medical College of Georgia, Augusta University, Augusta, GA, United States; ^3^University of Texas at Austin, Austin, TX, United States; ^4^Victoria University of Wellington, Wellington, New Zealand; ^5^Lega F. D’Oro Research Center, Osimo, Italy

**Keywords:** microswitch-aided programs, activity, multiple disabilities, stimulation, physical exercise

## Abstract

**Background:**

People with severe/profound multiple (e.g., intellectual, motor, or sensory–motor) disabilities are frequently restricted to a situation of inactivity and dependence, which may be modified by promoting functional activity engagement through assistive technology.

**Methods:**

This study assessed the possibility of promoting functional activity engagement *via* microswitch-aided programs with nine participants with multiple disabilities between 10 and 29 years of age. Functional activity consisted of constructive interaction with the immediate environment (e.g., reaching/touching or putting away objects) through the use of response schemes considered practical and beneficial for the participants’ physical exercise and general condition. Microswitch-aided programs were used to monitor the participants’ responses and to automatically provide stimulation opportunities contingent on those responses.

**Results:**

All participants had a large/significant increase in their activity engagement (i.e., response frequencies) during the microswitch-aided programs, when compared to the baseline periods. These data, which are in line with previous findings in the area, indicate that the programs targeted activity and responses suitable for the participants and ensured contingent stimulation effective to motivate them.

**Conclusion:**

People with severe/profound multiple disabilities can engage in functional activity with the help of microswitch-aided programs.

## Introduction

People with severe/profound multiple (e.g., intellectual, motor, or sensory–motor) disabilities are frequently restricted to a situation of inactivity and dependence (1–4). They typically lack the motor skills required for carrying out conventional occupational activities and may also be unable to control basic body schemes/responses necessary to reach and manipulate objects in their proximity (5, 6). Their motor disabilities, often aggravated by the presence of sensory (i.e., hearing or visual) impairment, may also imply that they cannot access and control environmental stimulation by themselves and depend on caregivers for their stimulation input (6–8). Such a motor or sensory–motor situation, combined with the people’s intellectual disabilities, makes any attempt to set up an intervention program a difficult endeavor. Indeed, one has to identify (a) functional and practical program objectives for the people and (b) effective and affordable ways to pursue those objectives (2, 9, 10).

In terms of objectives, one could envisage different forms of functional activity to be selected on the basis of the people’s general characteristics. One such form of activity could involve simple use of objects (e.g., placing objects into containers) (3, 4, 10). This form of activity would be considered functional for participants able to handle objects, as it entails (a) practical and meaningful interaction with the environment counteracting their passivity and improving their social image and (b) exercise of relatively elaborate response schemes with possible benefits for their overall physical condition (11–14). Another form of functional activity could involve the performance of response schemes typically targeted by physiotherapy, such as arm stretching to touch an object or moving the trunk forward (15). This form of activity would be considered functional for participants unable to handle objects, as it entails independent engagement in movement/exercise that counters passivity and may have relevant implications from a social and a physical rehabilitation standpoint (10, 16).

Ways of pursuing the aforementioned objectives in an effective, convenient, and affordable manner (i.e., sustainable within applied settings without heavy requests on staff time) necessarily include the use of assistive technology (6, 16–19). Assistive technology might be employed to monitor the participants’ responses in an automatic and reliable manner and could also serve to provide stimulation opportunities contingent on those responses, thus motivating the participants to strengthen and maintain their responding (6, 19–22).

Research studies have recently been reported, which targeted the aforementioned forms of functional activity and employed assistive technology (i.e., microswitch-aided programs) in the process (9, 10, 15, 16). The results of those studies were encouraging, but the number of participants involved was relatively small. Additional studies would seem warranted to extend the assessment and confirm the possibility of pursuing those forms of activity through new microswitch-aided programs with persons with severe/profound and multiple disabilities (23). This study served to extend the assessment by involving nine new participants, who pursued either the first or the second form of activity (i.e., either simple use of objects or performance of specific motor schemes) with the support of microswitch-aided programs.

## Materials and Methods

### Participants, Activity, and Responses

Table 1 lists the nine participants involved in the study, with their assigned pseudonyms, their ages and sensory disabilities, the type of activity selected for them, and the responses required. All participants had a diagnosis of encephalopathy due to congenital or perinatal causes and presented with extensive motor impairment, low or minimal vision and/or hearing loss, and severe/profound intellectual disabilities. The motor impairment of the first two participants (i.e., Steve and Mike) precluded walking but allowed some arm and hand control with the possibility of holding/manipulating objects. The motor impairment of the other seven participants (i.e., Luke, Fred, Ted, Alex, Sam, Lisa, and Andy) precluded walking and also hindered arm and hand movements with inability to hold/manipulate objects. The levels of intellectual disabilities had been estimated by the psychological services of the education and rehabilitation contexts that the participants attended. Yet, no formal testing or IQ scores were available due to the participants’ conditions.

**Table 1:** Participants’ characteristics, activity, and responses.

Participants	Ages (years)	Sensory disabilities	Activity type and responses
1. Steve	10	Minimal residual vision and moderate hearing loss	Type I. Take objects from the tabletop and put them in a container
2. Mike	21	Minimal residual vision and moderate hearing loss	Type I. Detach objects from a board and put them in a container
3. Luke	28	Minimal residual vision	Type II. Right arm stretching to reach and push a panel
4. Fred	29	Minimal residual vision	Type II. Left arm stretching to reach and move a bottle
5. Ted	29	Minimal residual vision	Type II. Right arm stretching to reach and push a panel
6. Alex	18	Minimal residual vision	Type II. Right arm stretching to reach and stroke a panel
7. Sam	15	Low vision	Type II. Right arm stretching to reach and stroke a multi-section panel
8. Lisa	23	Moderate-to-severe hearing loss	Type II. Right arm stretching to reach and stroke a panel; and trunk forward movements
9. Andy	20	Minimal residual vision	Type II. Right arm stretching to reach and push a panel; and left arm stretching to reach and push a panel

Steve and Mike were assigned an activity of the first type, and their responses consisted of collecting objects from the tabletop or a board and putting them into a container (see Table 1). The last seven participants were assigned an activity of the second type. For five of them (i.e., Luke, Fred, Ted, Alex, and Sam), the activity involved a single response, which consisted of stretching the right or left arm to reach and push/stroke a panel or move a bottle (see Table 1). For the last two participants (i.e., Lisa and Andy), the activity involved two different responses. Those responses concerned movements of the right arm or of the trunk for Lisa, and movements of the right or of the left arm for Andy (see Table 1).

Informal talks with families and staff had indicated that they supported the participants’ activity involvement *via* microswitch-aided programs and considered it (a) beneficial in terms of constructive occupation and physical exercise (with supposedly positive therapeutic consequences) and (b) also enjoyable (due to the stimulation available for activity engagement). The participants’ legal representatives had provided written informed consent for the study, which complied with the 1964 Helsinki Declaration and its later amendments and was approved by a relevant Ethics Committee.

### Setting, Technology, and Stimuli

The programs were carried out in a quiet room of the education and rehabilitation contexts that the participants attended. The technology entailed a computer with sound amplifier, which was connected to specific microswitches and equipped with basic software. The microswitches and computer served to (a) detect and record the responses targeted for the participants (i.e., during baseline and intervention sessions) and (b) trigger the delivery of brief periods of preferred stimulation contingent on those responses (i.e., during the intervention sessions). Table 2 lists the microswitches used for the different participants. The microswitches used for Steve and Mike, who had an activity of the first type, consisted of a modified touch sensor and an array of optic sensors, respectively (6). The touch sensor was activated any time Steve dropped into a container an object he had collected from the tabletop. An optic sensor was activated any time Mike detached an object from a board in front of him. The object was then to be dropped in a container under the board.

**Table 2:** Participants’ microswitches.

Participants	Microswitches
1. Steve	Touch sensor in the container in which the objects were to be placed
2. Mike	Optic sensors under the objects to be detached from a board and placed in a container
3. Luke	Pressure sensor attached to the panel he had to reach and push with his right hand
4. Fred	Tilt sensors attached to the bottle he had to reach and move with his left hand
5. Ted	Pressure sensor attached to the panel he had to reach and push with his right hand
6. Alex	Touch-sensitive pad attached to the panel he had to reach and stroke with his right hand
7. Sam	Touch-sensitive pad attached to the multi-section panel he had to reach and stroke with his right hand
8. Lisa	Touch and pressure sensors attached to the panel she had to reach and stroke with her right hand; and pressure sensors on her chair’s back that she had to free with her trunk forward movements
9. Andy	Pressure sensor attached to the panel he had to reach and push with his right hand or with his left hand

Pressure, touch, and tilt sensors were used as microswitches for the next seven participants (6). For Luke, Fred, Ted, Alex, and Sam, microswitch activation occurred any time they performed their target response (i.e., they pushed or stroked a panel or moved a bottle following right or left arm stretching) (Table 2). For Lisa and Andy, microswitch activation occurred as they performed the response being targeted at the time (see below). The response could be (a) stroking a panel following right arm stretching or lifting the trunk from the chair’s back (Lisa) and (b) pushing a panel following right arm stretching or left arm stretching (Andy) (see Table 2).

The stimuli that the participants could access with their responses (i.e., microswitch activations) included, among others, audio- or video-recordings with music and familiar voices, lights, airflows, and/or vibratory events. The stimuli had been recommended by staff and selected after preference screening. The screening process involved 15 or more non-consecutive presentations of brief segments of each of the stimuli available. A stimulus was selected for the study if the two research assistants who conducted the screening agreed that such a stimulus fostered participant’s positive reactions (e.g., alerting, orienting, or smiling) in 60% or more of the presentations.

### Experimental Conditions and Data Analysis

The experimental design used for all participants, except Lisa and Andy, was an ABAB, in which A and B represented baseline and intervention phases, respectively (24). The experimental design used for Lisa and Andy was a multiple probe across the two responses used for them (24). They started with baseline sessions on both responses. Then, they had (a) intervention sessions on the first response (i.e., right arm stretching to touch a panel), (b) new baseline and intervention sessions on the second response (i.e., trunk forward movements for Lisa and left arm stretching to touch a panel for Andy), and (c) post-intervention sessions on both responses.

The statistical significance of the response changes/increases from each baseline (A) to the subsequent intervention phase (B) of the first seven participants was assessed through the Kolmogorov–Smirnov test for two data sets (25). The same test was used to determine the significance of the changes occurring from the baseline to the intervention phase on each of the two responses of Lisa and Andy.

#### Baseline

During baseline sessions, the participants had their activity/technology arrangement, but received no stimulation for the responses.

#### Intervention

Conditions differed from baseline in that the participants received 10 s of preferred stimulation (i.e., 10 s of one or a combination of the stimuli selected for them) after each response occurrence. The stimulation was regulated by the computer system automatically. For Mike and Fred, about one-third of the stimulation instances would end up with a one- or two-word utterance, such as their “Name” or “Come on,” considered useful to support alertness and, possibly, responding (15). The first intervention phase (for participants with a single response) or the start of the intervention on each response (for participants with two responses) was preceded by four to six practice sessions in which prompting was available to help the participants rehearse their response and experience the stimulation following it (see below).

#### Post-Intervention

This phase was available only for Lisa and Andy. Conditions were as during the intervention, except that sessions with one response were alternated with sessions with the other response daily or every other day.

### Sessions, Response Recording, and Research Assistants

Sessions lasted 5 min for all participants (i.e., in line with the recommendations of staff and families) and were mostly carried out between three and eight times a day, over study periods of about 1–3 months. Differences in numbers of sessions across participants were mostly due to their availability. The responses, which brought about microswitch activations, were recorded by the computer system automatically. Five experienced research assistants were in charge of the sessions, using the technology and response prompting (i.e., verbal and physical guidance ensuring the participant’s response performance). Response prompting occurred (a) prior to the start of baseline and intervention sessions and (b) during those sessions if the participant did not emit any response independently for a period of 30–65 s (15). Response prompting was also used during the practice sessions, as mentioned earlier (i.e., to foster response and stimulation experience). Baseline and intervention responses that occurred with prompting were subtracted from the computer total. Agreement between research assistants on recording such responses was assessed in 25 sessions. In each of those sessions, the research assistants’ reported frequencies (which could also be 0) corresponded, thus indicating complete agreement.

## Results

**Figure 1:**
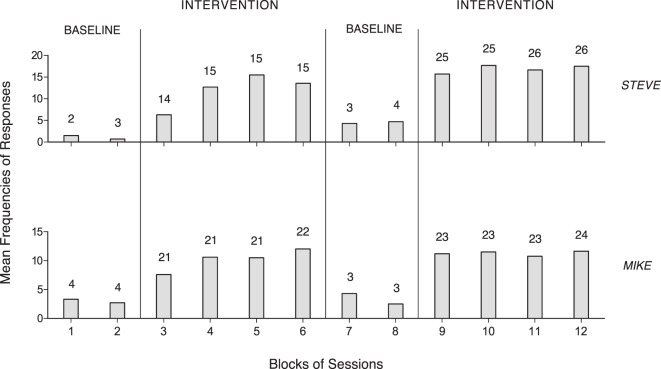
The two panels summarize the data for Steve and Mike. The bars represent mean frequencies of response occurrences per session, over blocks of sessions. The number of sessions included in each block is indicated by the numeral above it.

**Figure 2:**
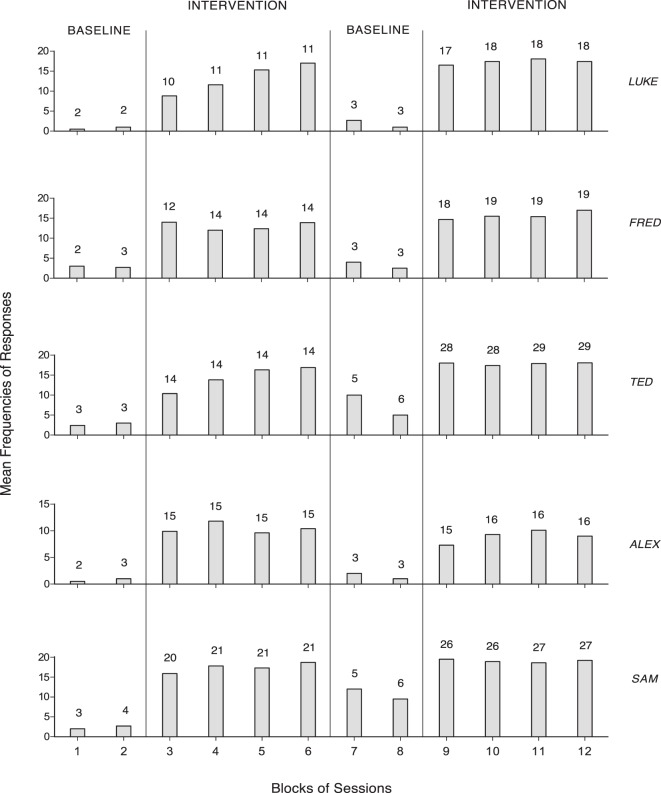
The five panels summarize the data for Luke, Fred, Ted, Alex, and Sam, which are plotted as in Figure 1.

**Figure 3:**
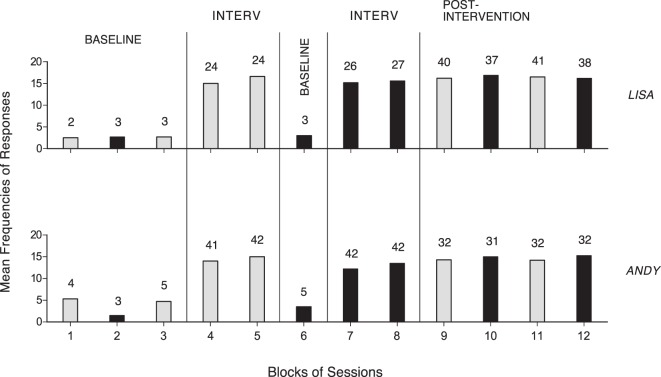
The two panels summarize the data for Lisa and Andy. The gray and black bars represent mean frequencies of occurrences per session for the participants’ first response (i.e., right arm stretching) and second response (i.e., trunk forward movements and left arm stretching), respectively. Mean frequencies are plotted as in Figure 1.

The two panels of Figure 1 summarize the data for Steve and Mike, who were provided with an activity of the first type. The five panels of Figure 2 summarize the data for Luke, Fred, Ted, Alex, and Sam, who had an activity of the second type, and used a single response. The two panels of Figure 3 summarize the data for Lisa and Andy, who had an activity of the second type and used two different responses. The gray bars of Figures 1 and 2 indicate mean frequencies of responses performed per session, over blocks of sessions. The number of sessions included in each block is indicated by the numeral above it. The gray and black bars of Figure 3 represent mean frequencies of occurrences per session for the participants’ first and second response, respectively. Those frequencies are computed over blocks of sessions, as in the previous figures.

Figure 1 shows that Steve and Mike had mean response frequencies of about one and three per session during the first baseline (five and eight sessions). During the first intervention phase (59 and 85 sessions), they reached means exceeding 12 and 10 responses per session, respectively. The second baseline (7 and 6 sessions) and the second intervention phase (102 and 93 sessions) showed a response decline and a new response increase, which confirmed the increase observed in the first intervention phase, respectively.

Figure 2 shows that Luke, Fred, Ted, Alex, and Sam had mean response frequencies not exceeding three per session during the first baseline (four to seven sessions). Their mean frequencies increased to between about 10 (Alex) and 17 (Sam) during the first intervention phase (43–83 sessions). The second baseline (6–11 sessions) and the second intervention phase (63–114 sessions) showed a response decline and a new response increase confirming the increase observed in the first intervention phase, respectively.

Figure 3 shows that Lisa and Andy started with mean baseline frequencies for the two responses ranging from below two to about five per session. The intervention on the first response (48 and 83 sessions) increased its mean frequencies to about 15 per session. The new baseline (three and five sessions) and the intervention (53 and 84 sessions) on the second response showed values similar to those reported for the first response. The post-intervention phase (156 and 127 sessions) showed that the mean response frequencies matched those observed during the intervention periods. The Kolmogorov–Smirnov test confirmed that the differences between baseline and intervention response frequencies were statistically significant (*p* < 0.01) for all nine participants (25).

## Discussion

The results indicate that all participants increased their response engagement irrespective of whether they were provided with an activity of the first type (i.e., Steve and Mike) or an activity of the second type (i.e., the other seven participants). Indeed, Lisa and Andy carried out their activity using two different responses. These results extend and corroborate previous data (9, 10, 15) and offer an encouraging perspective as to the possibility of using technology-aided programs to promote functional activity in persons with severe/profound intellectual and multiple disabilities. In light of these results, a number of considerations may be put forward.

First, the two types of activity seemed appropriate for (a) fostering the participants’ constructive engagement, (b) counteracting their passivity, and (c) helping them exercise practical/functional motor schemes with likely advantages for their physical condition (1, 3, 11–14, 26, 27). These forms of activity engagement could also be viewed as relevant supplements to conventional physiotherapy (10, 11), with consequent extension of the participants’ exercise time and possible benefits in terms of motor fluency, muscle tone, respiratory functions, and/or blood circulation (11–14, 16, 26, 28). Those supplements could also be much more enjoyable for the participants than the conventional physiotherapy given that the responses are followed by preferred stimulation (29–33).

Second, the generally positive outcome of the study may be attributed to three main conditions, that is, (a) the selection of responses that were feasible (albeit fairly demanding) for the participants, (b) the use of stimulation events that were preferred/enjoyable for the participants and thus may have acted as reinforcers, motivating the participants’ performance, and (c) the employment of program technology that was fitting the participants’ conditions and was adequate to the goals planned for them (6, 23, 29–33). The selection of responses can be conceived as a task, which requires the cooperation of regular staff and physiotherapists so as to avoid misjudgments and errors with negative consequences for the final outcome of the program (6, 16, 34). The selection of stimulation may need to involve a direct preference screening in addition to staff recommendations (35, 36). The technology may need to include (a) simple microswitches that can be applied easily and quickly and can monitor the participants’ responses in a reliable manner and (b) a stimulation delivery system that can activate various stimuli considered effective for the participants and allow session variations in terms of stimuli presented (6, 34, 37–39).

Third, while the stimulation seemed effective in increasing the response frequencies of all participants during the intervention phases, differences among participants were also visible (see, for example, Alex and Sam). The data available do not allow any specific answer on these differences. At present, only two general hypotheses might be formulated to explain them. One such hypothesis postulates differing levels of functioning across participants, with different behavioral abilities, degrees of alertness, and interest for environmental stimuli. Another hypothesis postulates differing levels of difficulty (relative cost) of the response and/or differing levels of impact (motivating power) of the selected stimuli (15, 29–31, 33).

Fourth, several limitations of this study may need to be pointed out here and remedied by new research. The first limitation is the relatively small number of participants involved in the programs and the consequent difficulty to determine the strength of the present findings and to shed some light on the performance differences across participants. New studies would need to extend the assessment, which should also include follow-up data (31, 40). The second limitation concerns the lack of any specific data regarding (a) the possible benefits of the participants’ response performance (exercise) and (b) the supposed enjoyment of such performance (15). With regard to the benefits, new studies could monitor a number of participants’ parameters, such as, heart rates, muscle tone, body fluids regulation, and sleep patterns (41). With regard to performance enjoyment, new studies could record the participants’ indices of happiness (e.g., smiles) during the sessions, thus extending earlier work in this area (6, 9, 32, 42). A third limitation is the lack of a social validation assessment directed at gathering the opinion of families and staff about the impact and adequacy of the program conditions (43, 44). Such an opinion could easily influence future decisions regarding adoption and generalization of those conditions. A fourth limitation of the study concerns the lack of reliability checks on the research assistants’ performance (i.e., using microswitches and prompting responses). Although such performance should be monitored carefully, the extended experience of the research assistants employed was thought to provide reasonable guarantee about their dependability.

In conclusion, microswitch-aided programs can represent a relevant resource for helping people with extensive multiple disabilities engage in functional activity (6, 20, 37, 38). New research would need to (a) remedy the aforementioned limitations of the present study, (b) identify additional forms of activity and responses, and (c) pursue technology updates that could make the intervention programs more widely and reliably applicable (15, 27, 34, 45, 46).

## Ethics Statement

Appropriate institutional board approval and written informed consent were obtained for the study. All procedures were in accordance with the ethical standards of the institutional and/or national research committee and with the 1964 Helsinki Declaration and its later amendments or comparable ethical standards.

## Author Contributions

GL, NS, MO, and JS were responsible for setting up the study, acquiring/analyzing the data, and writing/editing the manuscript. GA, VP, and FC contributed in acquiring and analyzing the data and editing the manuscript.

## Conflict of Interest Statement

The authors report no conflicts of interest. The authors alone are responsible for the content and writing of the article.
